# Highly Electroconductive Metal-Polymer Hybrid Foams Based on Silver Nanowires: Manufacturing and Characterization

**DOI:** 10.3390/polym16050608

**Published:** 2024-02-23

**Authors:** Petrică Linul, Radu Bănică, Oana Grad, Emanoil Linul, Nicolae Vaszilcsin

**Affiliations:** 1 Faculty of Industrial Chemistry and Environmental Engineering, Politehnica University Timisoara, Piata Victoriei 2, 300 006 Timisoara, Romania; 2 National Institute for Research and Development in Electrochemistry and Condensed Matter, Dr. A. Paunescu Podeanu Street, No. 144, 300 569 Timisoara, Romania; 3 Research Institute for Renewable Energy, Politehnica University Timisoara, 138 Gavril Musicescu, 300 501 Timisoara, Romania; 4 Department of Mechanics and Strength of Materials, Politehnica University Timisoara, 1 Mihai Viteazu Avenue, 300 222 Timisoara, Romania

**Keywords:** silver nanowires, metal-polymer hybrid foams, electrical resistance, compression, microstructure

## Abstract

Due to their electroconductive properties, flexible open-cell polyurethane foam/silver nanowire (PUF/AgNW) structures can provide an alternative for the construction of cheap pressure transducers with limited lifetimes or used as filter media for air conditioning units, presenting bactericidal and antifungal properties. In this paper, highly electroconductive metal-polymer hybrid foams (MPHFs) based on AgNWs were manufactured and characterized. The electrical resistance of MPHFs with various degrees of AgNW coating was measured during repeated compression. For low degrees of AgNW coating, the decrease in electrical resistance during compression occurs in steps and is not reproducible with repeated compression cycles due to the reduced number of electroconductive zones involved in obtaining electrical conductivity. For high AgNW coating degrees, the decrease in resistance is quasi-linear and reproducible after the first compression cycle. However, after compression, cracks appear in the foam cell structure, which increases the electrical resistance and decreases the mechanical strength. It can be considered that PUFs coated with AgNWs have a compression memory effect and can be used as cheap solutions in industrial processes in which high precision is not required, such as exceeding a maximum admissible load or as ohmic seals for product security.

## 1. Introduction

The use of silver nanowires (AgNWs) is growing in a wide range of fields due to their exceptional properties and benefits in various technological applications, such as transparent electrodes [1], flexible conductors [2,3,4], pressure sensors [5], air conditioning filtration systems [6], controlled drug delivery devices [7], and antimicrobial dressings [8]. AgNWs have excellent electrical conductivity, making them ideal for use in touch screens. They ensure efficient transmission of touch signals, thus improving the performance and accuracy of displays. They also help to increase the durability of touchscreens, resisting wear and damage better than other materials [9,10].

The use of AgNWs in air conditioning filtration systems brings numerous benefits due to their antibacterial, antiviral, and antifungal properties. AgNWs have strong antibacterial and antiviral properties, helping to eliminate and prevent the growth of bacteria and viruses in the air filtration system. The antifungal properties of AgNWs help to prevent the formation of mold and fungi in the air filter, ensuring a healthier and cleaner environment [11,12]. Biofilm, a thin film formed by bacteria on filter surfaces, can affect the efficiency of the filtration system. The antibacterial properties of AgNWs can help reduce biofilm formation and growth. AgNWs have strong antimicrobial properties, preventing the growth and multiplication of bacteria in water filtration systems. This helps to keep the water clean and prevent microbial contamination [13,14]. Wang et al. [15] manufactured a conductive polymer composite-based strain sensor. They showed its unique advantages and the performance of the proposed sensor.

The manufacturing process of hybrid metal-polymer foams can combine some of the properties of these two distinct classes of materials. The process is based on mixing metallic particles with a polymer base, which can be epoxy, polyurethane [16], or other types, and molding into the desired shape, resulting in new materials with different mechanical, chemical, and electrical properties. Obtaining harder hybrid foams can be achieved by impregnating a metallic foam with a polymer [17] either by spraying droplets of melted metal [18] or by sintering metallic particles [19]. Impregnation can be performed under pressure or by using vacuum, depending on the size and nature of the pores of the metallic skeleton. These materials can subsequently undergo mechanical processing. Thus, parts can be obtained with increased mechanical and anticorrosive resistance for use in converting kinetic energy into heat such as in the automotive industry for passenger protection in the event of a collision [20].

Tammaro et al. [21] presented an innovative method of coating PLA filament with a liquid layer before printing by passing this filament through a bath. If this bath were made from a suspension of AgNWs, the printed piece would have intrinsic bactericidal properties due to the silver in the composition, with the presence of nanowires also increasing the electrical conductivity of the polymer, which would have better properties for discharging static energy. In ref. [22], a method of direct printing of porous foams through the generation of CO_2_ microbubbles in situ, right during the printing process, was presented, achieving a very low polymer density of just 40 kg/m^3^. It is predictable that if this polymer mass contained AgNWs, which are quasi-one-dimensional formations, during the bubbles’ expansion in the molten mass, a controlled flow of the liquid polymer mass would occur that would generate an ordering of the AgNWs, which could become parallel to the cell walls. Ordering of the AgNWs during printing could lead to the acquisition of special optical properties of the printed polymers, if the material is transparent to visible light. From our observations, simple homogenization of the nanowire suspension by unidirectional rotation of the dispersion medium leads to the achievement of specific optical effects, which are correlated with the one-dimensional nature of the wires.

Of course, if the goal is not to obtain foams with bactericidal properties, cheaper materials like carbon can be used instead of silver to produce electroconductive composites. Li et al. [23,24] fabricated electroconductive composite foams based on poly(ether block amide) (PEBA) and a carbon nanostructure (CNS), demonstrating their excellent electrical sensitivity to compression. The electrical conductivity could be significantly altered by changing the foam’s expansion ratio. As expected, an increase in the foam’s porosity leads to lower conductivity due to the reduced density of electric current pathways. The conductivity of the materials almost linearly increased with the addition of more carbon into the composite due to the increased density of electric current pathways [23]. A material with a similar composition showed good properties for electromagnetic interference (EMI) shielding, which changed with humidity, making these materials useful for manufacturing motion sensors attached to the human body [24].

Silver nanowire foams are a special class of materials that combine the structural properties of foams with the advantages of AgNWs. These foams can be used in various fields due to their distinct characteristics. Due to the conductive properties of AgNWs, the foams can provide a rapid response to pressure changes, allowing accurate detection of minute pressure variations. The use of AgNWs gives sensors chemical and mechanical stability over time, reducing their deterioration or degradation under long use [25,26,27,28]. Due to the combination of porous properties and AgNWs, the foams can be used in the production of sensors and medical devices, such as smart dressings or controlled drug delivery systems [29,30,31]. The antibacterial properties of silver help to prevent infection, and the porous structure of foams promotes the absorption of exudate from wounds. Porous foams can be used to encapsulate drugs, and AgNWs can provide antibacterial properties. Thus, these foams are used in controlled drug delivery systems, providing effective treatment and reducing the risk of infection [32,33,34,35].

The literature reports several works on the development and characterization of AgNWs foams. Wu et al. [36] developed a new class of graphene/AgNW foams using the polymer template-assisted assembly technique. The polymer template-assisted assembly technique is a simple, scalable, and low-cost strategy beneficial for large-scale production of hybrid foams. Lightweight and flexible waterborne polyurethane (WPU)/AgNW nanocomposites with micrometer-sized unidirectional pores were fabricated by Zeng et al. using the ice template freeze-drying method [37]. Also, Ahmed et al. [38] manufactured flexible, stretchable, and compressible piezoresistive sensors based on AgNWs using porous substrates with a negative Poisson’s ratio (auxetic foams). Auxetic foam sensors (AFSs) exhibited improved piezoresistive sensitivity compared to conventional foam substrates (CFSs) in tension and compression modes, with improvements of up to 290% and 165% (in terms of measurement factor), respectively. A flexible 3D macroporous conductor with high electrical conductivity, consisting of a poly(3,4-ethylenedioxythiophene):poly(styrenesulfonate) (PEDOT:PSS)/AgNWs composite coated on a commercial foam, was developed by Moon et al. [39]. The fabricated foam conductor exhibited high electrical conductivity with good stability under bending deformation conditions. Recently, Wan et al. [40] fabricated bacterial cellulose (BC)/AgNW composites as novel wound dressings through an innovative stepwise in situ biosynthesis. The BC/AgNW wound dressings exhibited abundant pores and adequate water vapor permeability, water absorption rate, and water retention rate, which were advantageous for reducing exudates and maintaining a moist environment.

Silver metallic foams, without any other additives, can be used as antibacterial agents for water purification or as catalysts for the conversion of CO_2_ to CO [41,42] in batteries, fuel cells, or supercapacitors [43]. Foams made of pure silver have low elasticity and their electrical resistance in the uncompressed state (i.e., native, the sample coated with AgNWs before any deformation through compression) has a very low value, comparable to that of the electrical conductors with which they are connected in the circuit. Therefore, determining the very small variation in electrical resistance depending on the degree of compression becomes difficult. In order to solve this problem, the microstructure (before and after test) and electrical behavior under repeated compression of AgNW-coated PUF was investigated for the first time, obtaining elastic hybrid materials (metal-polymer hybrid foams) with good properties. Newly developed materials combine both the advantages of PUFs (flexibility) and metallic AgNWs (high conductivity), resulting in elastic materials with sufficiently high native electrical resistance to be easily integrated into pressure sensors or filters with chemical and thermo-bactericidal properties.

## 2. Materials and Methods

### 2.1. Silver Nanowire Manufacturing

The aim was to obtain silver nanowires (AgNWs). Silver has the highest electrical resistivity of all metals in the solid state of aggregation, 1.59 × 10^−8^ Ωm, and the metal is non-toxic and hardly oxidizable. When it is in the form of micro- and nanoparticles, it can be used to make smart fabrics with integrated electrodes for biomedical applications [44], such as electrocardiograms [45], electromyography [46], or electroencephalography [47].

The synthesis of AgNWs was performed by dissolving 255 mg of silver nitrate (99.8% AgNO_3_, Sigma-Aldrich, St. Louis, MO, USA) in 30 mL of ethylene glycol (99% EG, Sigma-Aldrich), which was initially preheated at 150 °C for 10 min and then cooled to room temperature. Into this solution was added 15 mL of solution containing 249 mg poly-vinylpyrrolidone (PVP-40, Sigma-Aldrich) dissolved in a solution consisting of 15 mL of EG and 3 mL of 50 mM potassium chloride (KCl, Sigma-Aldrich) in EG solution. After the addition of KCl, the resulting solution became opalescent due to the formation of silver chloride (AgCl) nanocrystals [48].

Subsequently, the suspension was divided in two and transferred to two PTFE-lined (polytetrafluoroethylene/Teflon) autoclaves. In autoclave 1, 24 mL of the obtained solution was added. It was heat treated in the oven at 160 °C for 2 h 30 min. Further, the autoclave was allowed to cool to room temperature, after which the AgNWs were purified by centrifugation and washed five times in 96% ethanol (Chimreactiv, Ion Creanga, Nemt, Romania) (centrifugation was performed after each wash, followed by removal of the supernatant and washing with ethanol). Following centrifugation and ethanol rinsing, the AgNWs were placed in a 30 mL bottle and kept as a suspension in ethanol, with the headspace occupied by air. For autoclave 2, the same steps were followed, with the exception of the duration of the heat treatment, which was extended to 12 h. The silver concentration was determined thermogravimetrically by adding ethanol to the suspension until a concentration of 2 g/L AgNWs was reached. Finally, samples A1 and A2 were obtained from autoclaves 1 and 2, respectively.

### 2.2. Metal-Polymer Hybrid Foam Manufacturing

Flexible open-cell polyurethane foam (PUF) was used to obtain metal-polymer hybrid foams (MPHFs). The investigated PUF was a commercial foam with dimensions 120 mm × 75 mm × 30 mm (Jinhua, China). Using a hollow punch, foam cylinders with a diameter of 12 mm and height of 15 mm were obtained. Based on the mass and geometric parameters of the six samples, it was found that the PUF had a density of 18 ± 0.34 kg/m^3^. The samples that presented a standard deviation of the density greater than 3% were replaced.

**Figure 1:**
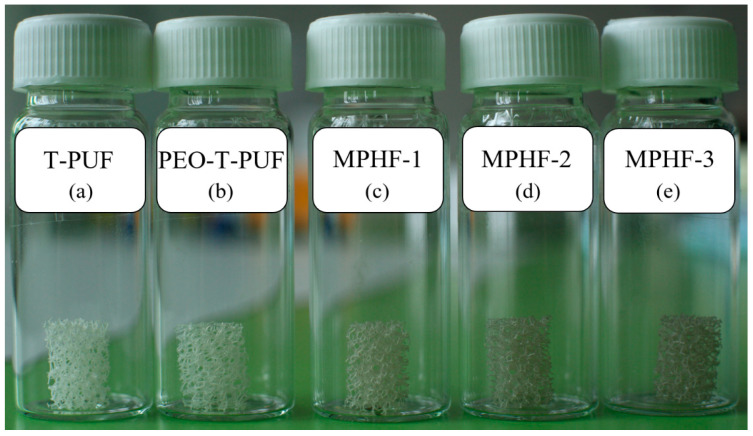
The T-PUF (**a**), PEO-T-PUF (**b**), MPHF-1 (**c**), MPHF-2 (**d**), and MPHF-3 (**e**) samples under investigation.

First, to wash the existing impurities (e.g., dirt, oils) from the manufacturing process, the obtained cylinders were immersed in ethanol preheated to 60 °C for 30 min, after which they were dried in an oven at 70 °C for 5 min. Thus, the first category of samples was obtained, named treated polyurethane foam (T-PUF), see Figure 1a.

In order to activate the hydrophobic surface of the PUF, the cylinders were immersed for 5 min in a 1:1 (volume ratio) ethanol–water solution (50% distilled water and 50% ethanol) of 0.1% poly(ethylene oxide) (PEO). The next step was to remove the droplets using compressed air, followed by drying in an oven at 70 °C for 5 min. The solubility of PEO in water was higher than in ethanol, yet ethanol wet the hydrophobic surface of PUF better, due to the fact that it contained both polar and nonpolar groups, both of which could interact with the polar and nonpolar groups on the surface of PUF. Additionally, the surface tension of ethanol is lower, facilitating wetting. A 1:1 ethanol–water mixture was prepared to obtain a PEO solution that allowed for wetting of the PUF surface. After the solvent evaporated, the ethanol–water vapor mixture could be easily condensed and recirculated in the process, making the process more economical and environmentally friendly. The immersion procedure was repeated two times, with the immersion time being 30 s. After the last immersion, the cylinders were dried in the oven at 130 °C for 10 min, obtaining the second category of samples (PEO-T-PUF), see Figure 1b.

After drying, a PEO-coated cylinder was immersed once for 20 s in an AgNW suspension of concentration 2 mg Ag/mL. Next, the sample was oven-dried on filter cloth at 130 °C for 5 min, so that the excess nanowire suspension that drained from the foam surface could be absorbed by it. Thus, sample MPHF-1 was obtained (see Figure 1c), belonging to the third category.

The same procedure was followed to obtain samples MPHF-2 (two immersions, see Figure 1d) and MPHF-3 (three immersions, see Figure 1e), from the fourth and fifth categories. So, in total, five types of samples were obtained (T-PUF, PEO-T-PUF, MPHF-1, MPHF-2, and MPHF-3), which were later investigated.

### 2.3. Experimental Setup

Initially, to determine the mechanical behavior, the PUFs were tested quasi-statically in compression. The experimental tests were performed on the electromechanical MTE-1L testing machine with a maximum load cell capacity of 50 N. The compression tests were performed at room temperature under displacement control using a test speed of 10 mm/min.

All manufactured samples (T-PUF, PEO-T-PUF, MPHF-1, MPHF-2, and MPHF-3) were characterized by scanning electron microscopy (SEM) using a Quanta FEG 250 (FEI, Hillsboro, OR, USA) equipped with an energy-dispersive X-ray (EDX) spectrometer. The heat treatment of the AgNW solutions and the obtained samples was performed in a preheated oven (Memmert UN 55, Schwabach, Germany). The AgNWs were purified by repeated centrifugations using a centrifuge (Sigma 3-30 KS, Osterode am Harz, Germany). After each centrifugation, the AgNWs were washed with ethanol and subjected to ultrasonication in order to purify the AgNWs using an ultrasonic bath (Raypa UCD-400, Barcelona, Spain).

**Figure 2:**
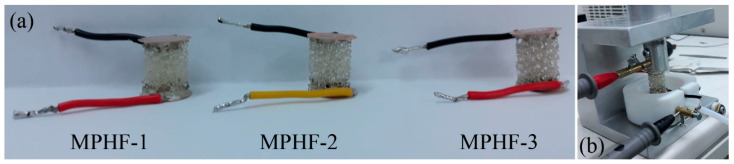
Sample preparation (**a**) and electrical resistance measurement setup (**b**).

In order to be able to measure the electrical resistance during the compression tests, it was necessary to prepare copper discs (14 mm diameter and 0.4 mm thickness) by grinding with alumina paste, upon which copper wires were tinned. These copper discs were used to ensure contact with the porous surface of the AgNW-coated samples. Contact between the samples and copper discs was obtained with the silver paste (Figure 2). Prior to measuring the electrical resistance, the samples were oven-dried at 50 °C for 14 h. Before starting the compression tests, the copper discs were short-circuited using a copper wire. After tightening the screws on the measuring instrument loops and fixing the set-up, the electrical resistance was measured, reaching a value of 0.08 Ω, which represented the resistance of the wires and all contacts. Before the compression test began, the shorting wire was cut, thus reading the total resistance of the assembly. The sample resistance was obtained by subtracting 0.08 Ω from the measured values. A resistance meter was used to measure the electrical resistance during compression.

## 3. Results and Discussion

### 3.1. Silver Nanowire (AgNW) Characterization

**Figure 3:**
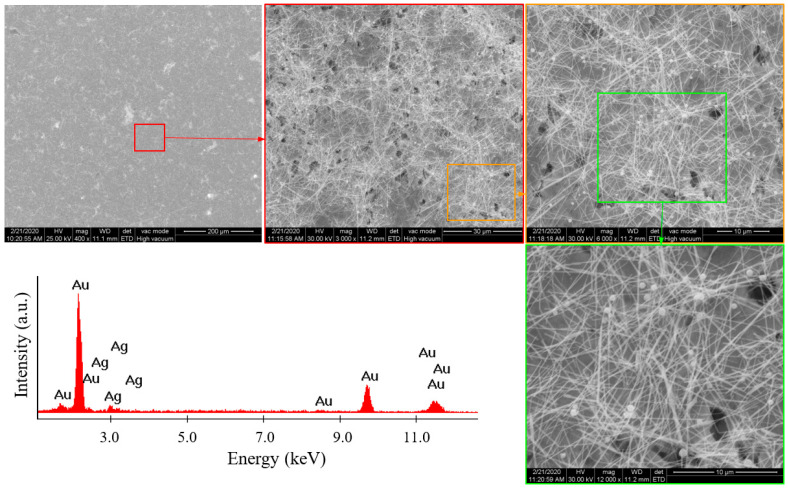
SEM images and EDX spectrum for the A1 sample (autoclave 1).

**Figure 4:**
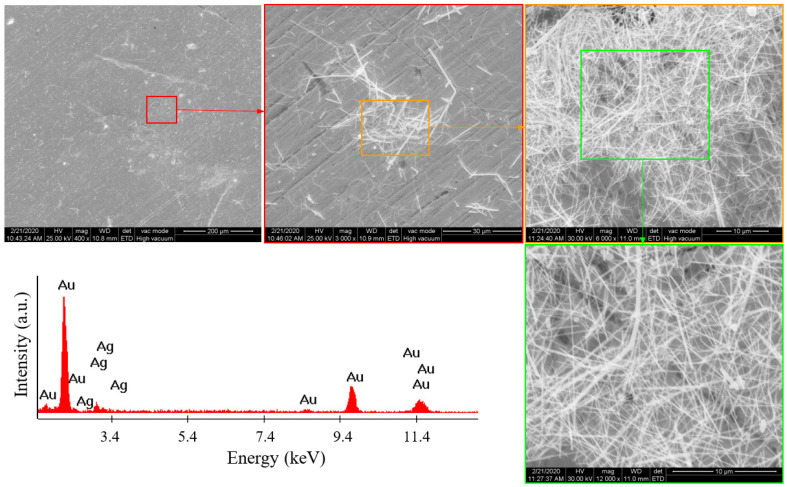
SEM images and EDX spectrum for the A2 sample (autoclave 2).

The obtained silver nanowires were characterized by SEM and EDX. For the SEM analysis, an acceleration voltage between 20 and 30 kV was used. To achieve good contrast between the nanowires and the PUF substrate, the samples were not metallized before the SEM analysis. For the micrographs of foams without AgNWs, the large field detector (LFD) was used in low vacuum mode to discharge the static energy of non-conductive samples. For foams coated with AgNWs, the backscattered detector (BSED) was used, which was sensitive to the atomic mass number, achieving good contrast between the foam and the metallic nanowires. And for the samples deposited on gold, the Everhart–Thornley detector (ETD) in high vacuum mode was used, which provided surface information, rendering the fine texture of the sample at high magnification. For the EDX analysis, acceleration voltages of 25 and 30 kV were used, which allowed for the electronic transitions of all elements present in the sample, enabling their identification. The SEM analyses and the EDX spectra are presented in Figure 3 and Figure 4 for the two previously mentioned samples (A1 and A2).

From the SEM image analysis, it can be seen that the AgNWs in sample A1 (obtained in autoclave 1) were longer (30 µm) than those in sample A2 (20 µm). It was also found that the diameter of the AgNWs in the A1 sample was smaller than that in the A2 sample. Moreover, the A1 sample showed a more uniform dispersion of AgNWs. The presence of gold in the EDX spectra was due to the gold support on which the A1 and A2 samples were placed. All other peaks belonged to silver.

In the case of sample A2, the duration of the solvothermal heat treatment was about five times longer than that of sample A1. With the increase in synthesis time, AgNWs grew in length and thickness through a recrystallization process, as shown in our research. This process was due to the dissolution of silver particles with other shapes and thin AgNWs due to the oxygen present in the empty space of the autoclave. The influence of oxygen traces on the selective dissolution of silver nanoparticles is well known in the literature. This particle growth process through crystallization is called Ostwald ripening, which is based on reducing the system’s entropy. The reactions that can occur are as follows:

4Ag_(nanoparticles, NWs)_ + O_2(dissolved)_ → 2Ag_2_O (1)

2Ag_2_O ↔ 4Ag^+^ + 2O^2−^ (2)

Ag^+^ + e^−^ → Ag^0^_(on NW)_ (3)

At the same time, as the synthesis time increased, the purity of the AgNWs increased, but thick AgNWs were less flexible than thin ones. For a uniform coating with AgNWs on PUF, thin AgNWs were needed, preferably with a length smaller than the smallest radius of the curvature of the PUF pores, so that they could coat the curved surfaces of the cell walls without bending. The SEM images showed that the smallest radius of the curvature of the foam pores was about 100 μm, making thin AgNWs with an average length of 8–30 μm suitable for this application. Nanowires with much shorter lengths ensured a more uniform coating, but the electrical conductivity of the foam would be more significantly affected by the numerous contact points between wires. This aspect will be discussed further. Therefore, due to its properties (flexibility, length, diameter, and dispersion), sample A1, containing thinner AgNWs, was preferred for the PUF coating experiments.

### 3.2. Flexible Open-Cell Polyurethane Foam (PUF) Characterization

**Figure 5:**
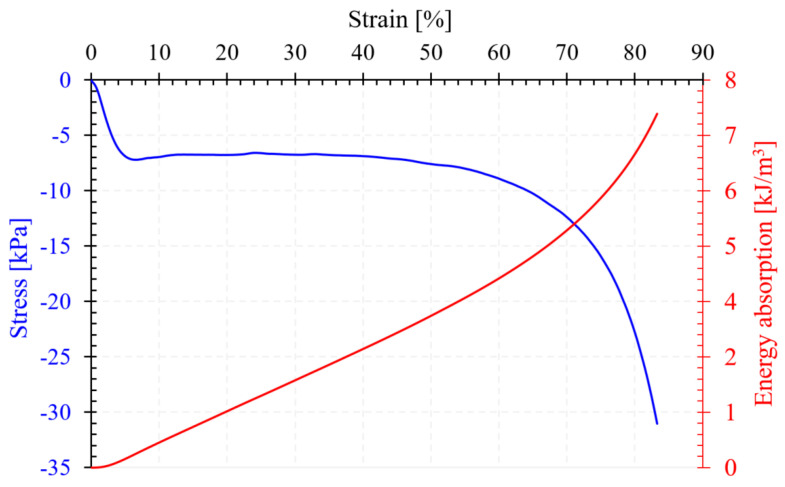
Compressive stress–strain and energy absorption–strain curves of PUF.

The PUFs were mechanically characterized by quasi-static compression tests. Figure 5 shows the characteristic stress–strain and energy absorption–strain curves of the investigated PUF. The presented curves are the most representative of those resulting from the experimental tests. In order to obtain reproducibility of the results, six cubic specimens were tested in compression. The stress–strain curve was typical of cellular materials with three characteristic zones, namely linear-elastic, plateau, and densification (see the blue curve in Figure 5) [49]. Each zone defined specific compression properties [50].

A compressive modulus of 203.09 kPa was found in the linear-elastic zone. This relatively short zone, at a strain below 6%, ended with the first maximum point, at which the compression strength (6.69 kPa) was identified. Associated with this point, obtained at the first increase in strain without an increase in stress, the strain at strength (5.74%) was determined.

**Figure 6:**
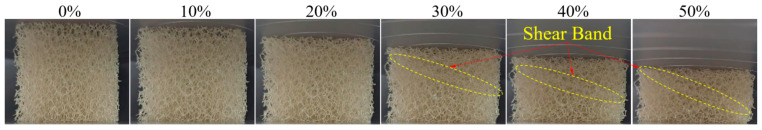
Compressive deformation sequences of PUF.

Beyond this point, the second region developed with a long and almost horizontal plateau. The specific characteristic of this region was the plateau stress, with a value of 6.51 kPa. The plateau stress was obtained as the arithmetic mean between the stresses corresponding to strains of 20 and 40% [51]. This area was closely related to the failure of the specimen and the collapse mechanisms that occur at the microstructural level [52]. To understand this aspect, Figure 6 shows the deformation sequences of the sample at different strain levels. It was found that the main failure mechanism in the tested PUF samples was the appearance of the shear band. This band was defined by a more pronounced compaction of the foam cells and the appearance of a whitish area (see the area highlighted in yellow in Figure 6) [53].

The plateau region ended with the beginning of the densification region (>55% strain). The characteristic point between these regions was defined by two properties: densification stress (8.28 kPa) and densification strain (57.18%).

The absorbed energy increased almost linearly with the compressive strain [54], with a slightly strengthened slope after 60% strain (see the red curve in Figure 5). Energy absorption at densification showed a value of 3.72 kJ/m^3^. On the other hand, the energy efficiency of the samples showed a maximum mean value of 45.02%.

It was observed that after the completion of the compression test, the specimen presented a quite pronounced height recovery (over 93%). However, over an hour after completing the test, the recovery proved to be even greater (almost complete).

### 3.3. Metal-Polymer Hybrid Foam (MPHF) Characterization

#### 3.3.1. SEM Analysis

The purpose of the SEM analysis was to observe/investigate the surface of the treated foams (T-PUF and PEO-T-PUF) as well as the dispersion of AgNWs on the foam walls (MPHF-1, MPHF-2, and MPHF-3).

**Figure 7:**
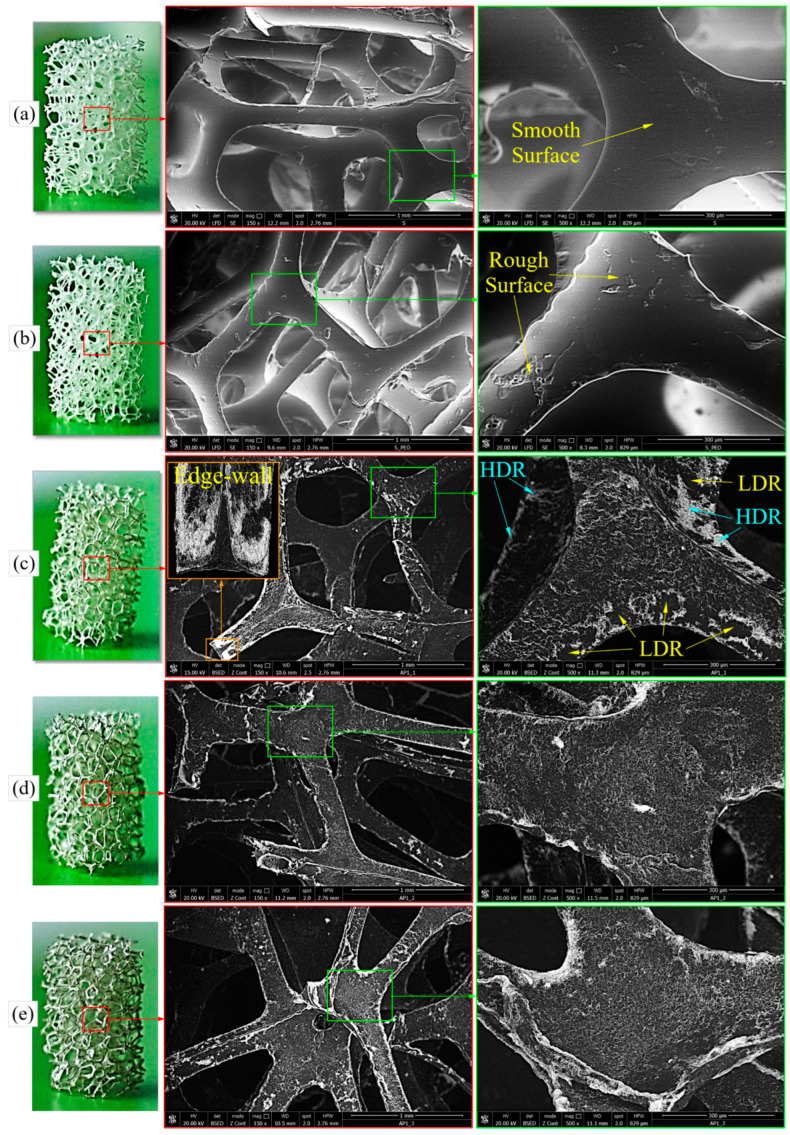
Images for T-PUF (**a**), PEO-T-PUF (**b**), MPHF-1 (**c**), MPHF-2 (**d**), and MPHF-3 (**e**) samples.

Polymeric foams are found with open, closed, or mixed (partially closed and partially open) pores/cells [55,56]. For the largest active surface area, a foam with open pores was chosen [57]. Polymer-based open-cell foams consist of an interconnected network of solid edges with well-defined pore shapes [58]. More precisely, open-cell foams contain solid material only at their edges, with the cells separated by open windows [59]. The chosen PUF had a cell size of about 1.2 mm and a cell wall thickness of 250 µm. Figure 7 shows macroscopic and microscopic images of the investigated samples.

The foam treated in ethanol (T-PUF) showed a smooth surface of the pore walls (see Figure 7a).

As can be seen in Figure 7b, PEO predominantly attached to the foam wall edges. The surface of the PEO-T-PUF sample walls was rougher compared to that of T-PUF. This aspect was due to the adhesion of PEO to the surface of the foam. The purpose of treating T-PUF with PEO was to achieve the best adhesion of AgNWs to the surface of the foam walls.

The optical and SEM images of the MPHF-1 (Figure 7c), MPHF-2 (Figure 7d) and MPHF-3 (Figure 7e) samples confirmed the deposition of AgNWs on the entire surface of the PEO-coated foams, with predominant deposition on the edges of the foam walls. It can be observed that the layer of AgNWs deposited on the PUF surface was quasi-two-dimensional, without significant three-dimensional porosity. This was due to the high aspect ratio of the pentagonal AgNWs, which, after the drying of the dispersion medium, were in the same plane as the PUF surface plane. Only in areas where there were significant agglomerations of AgNWs could some three-dimensional pores generated by the stochastic arrangement of the wires be observed. However, more low density regions (LDRs) and high density regions (HDRs) could be observed on the sample walls in the case of sample MPHF-1. These regions were difficult to identify in the case of samples MPHF-2 and MPHF-3. Moreover, from the optical images it can be seen that the color of the samples changed from slightly silvery tint in the case of MPHF-1 to a stronger silver tint for MPHF-3. It seemed that multiple immersions in the AgNW suspension led both to a more pronounced silver tint and to a uniform deposition of AgNWs.

#### 3.3.2. Electrical Behavior

**Figure 8:**
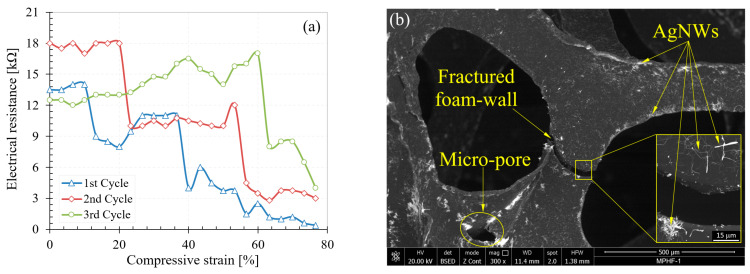
Variation of electrical resistance (**a**) and the MPHF-1 sample after testing (**b**).

**Figure 9:**
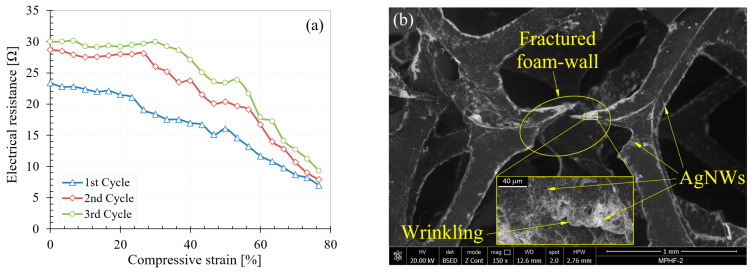
Variation in electrical resistance (**a**) and the MPHF-2 sample after testing (**b**).

**Figure 10:**
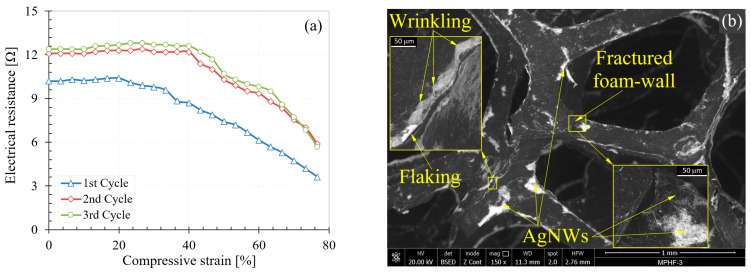
Variation in electrical resistance (**a**) and the MPHF-3 sample after testing (**b**).

Initially, AgNW deposition tests were also performed without applying the PEO layer on the polyurethane foam surface. No deposition of AgNWs was observed on the foam surface without the PEO layer. The electrical resistance of the foam without PEO was measured and the measurement showed that the samples were perfectly insulating. Subsequently, the electrical behavior of the MPHF-1, MPHF-2, and MPHF-3 samples was investigated during compression. Graphical representations of electrical resistance as a function of degree of compression for all samples are presented in Figure 8, Figure 9 and Figure 10. The compression test was carried out following three load cycles for all samples. The cycle number was associated with the number of sample loads. For example, for cycle 3, the measurement of the electrical resistance was performed during the 3rd compression load. For all cycles, the loading of the sample was carried out up to a strain of 80%.

From Figure 8a, it can be seen that the resistance of the MPHF-1 sample during compression remained relatively constant across the different strain levels, then dropped sharply between them. Overall, the electrical resistance decreased with the increase in the strain level. However, for cycle 3, a progressive increase in resistance was observed up to a strain of 60%, followed by a sudden decrease in the 60–80% range. This phenomenon was due to the agglomeration of AgNWs on the cell wall edges. This slight increase in resistance for sample MPHF-1 in cycle 3 between 0–60% compressive strain was probably due to the decrease in the number of contact points between the AgNWs, which was caused by the twisting of the PUF before the electrical resistance dropped abruptly upon touching the foam struts. In Figure 8b, both the dispersion of AgNWs on the surface of the pore walls and the brittle fracture of the walls could be easily observed. Also, some pores could be identified in the microstructure of the foam.

The nanowires in agglomerates had high conductivity, but the agglomerations of AgNWs were joined together by areas of low AgNW density, which determined the total resistance. During compression, new cell walls touched each other, causing the electrical resistance to drop sharply. The fact that this resistance decreased abruptly and not gradually, although the cell walls touched each other gradually, indicated that, in this sample, not all of the cell walls were coated by the AgNWs. Only when two cell walls coated with AgNWs touched each other did the electrical resistance decrease, whereas touching a cell wall not coated with AgNWs or a wall coated with an uncoated wall did not lead to a decrease in resistance. It can be seen that the resistance-compression curves for the three cycles were different, which may have been due to the fracturing of some of the AgNW-coated cell walls with the inherent disruption of the electrical path but also to the local destruction of the AgNW layer at the contact points of the cell walls.

The errors in measuring the electrical resistance were determined by the measurement multimeter’s error, which was approximately ±1% of the measured value, and by the electrical resistance of the copper wire with a length of about 40 mm used for short-circuiting. This had a calculated electrical resistance of 0.672 × 10^−3^ Ω. By short-circuiting the foam, it was possible to obtain the electrical resistance of the measuring loops and electrical contacts, which had a value lower than 0.1 Ohms (Ω), and this was subtracted from the read values. The sudden variation in electrical resistance was not due to measurement errors but rather to the effect of changing the number of electrical contact points between the metallic nanowires, as previously mentioned. In the case of covering PUF with a single layer of AgNWs, the influence of the number of electrical contact points between AgNWs on the total electrical resistance had a greater impact than the percolation process. Thus, at low AgNW coverage levels, the electrical resistance values in various compression cycles could not be theoretically identical because the contact points between AgNWs changed during each compression, always being different. Reproducible deformation of the cellular walls did not lead to reproducible electrical measurements in the absence of the percolation process, as observed from the experimental data for sample MPHF-1 for the first three compression cycles (see Figure 8a). With complete coverage of the cellular walls with AgNWs, every touch of the cellular walls decreased the length of the electrical pathway. At higher coverage levels, the reproducibility of electrical measurements increased because the elastic walls of the pores deformed in a similar and reproducible manner, with reproducible deformation translating into the reproducibility of the percolation process and implicitly of the measured electrical resistance values, as observed in Figure 9a.

In the case of the other two samples (MPHF-2 and MPHF-3), the variation in resistance showed less sudden decreases, which showed better homogeneity in the distribution of the AgNWs (see Figure 9a and Figure 10a). In other words, if a cell wall broke during compression, a new one partially took its place in the current conducting effect during compression.

For foams immersed two (MPHF-2) and three (MPHF-3) times, respectively, in the yarn superstructure, a plateau was clearly observed, where the strength remained constant up to a compression degree of 30–40% in cycles 2 and 3, followed by a quasi-linear decrease for higher compression degrees. A possible explanation for this phenomenon could be a phenomenon of partial disappearance due to mechanical micro-shocks or re-arrangement of the AgNWs in areas of strong bending of the cell walls during the first compression cycle, leading to a decrease in electrical resistance predominantly in those areas.

During the other compression cycles, as the density of the AgNWs in those areas was not influenced by the bending process of the walls, the electrical resistance would decrease only due to the mutual touching of the walls and not due to their bending. This theory will be evaluated in the future by SEM studies of the bent samples. If the theory is confirmed, the samples could be improved by adding an elastic polymer to the AgNW layer, which will return them to their original position after the compression–relaxation cycle is complete. For the MPHF-2 (see Figure 9b) and MPHF-3 (see Figure 10b) samples, in addition to the fracturing of the pore walls, flaking and wrinkling of the AgNWs appeared, a phenomenon that was not encountered in the case of the MPHF-1 sample (see Figure 8b).

**Figure 11:**
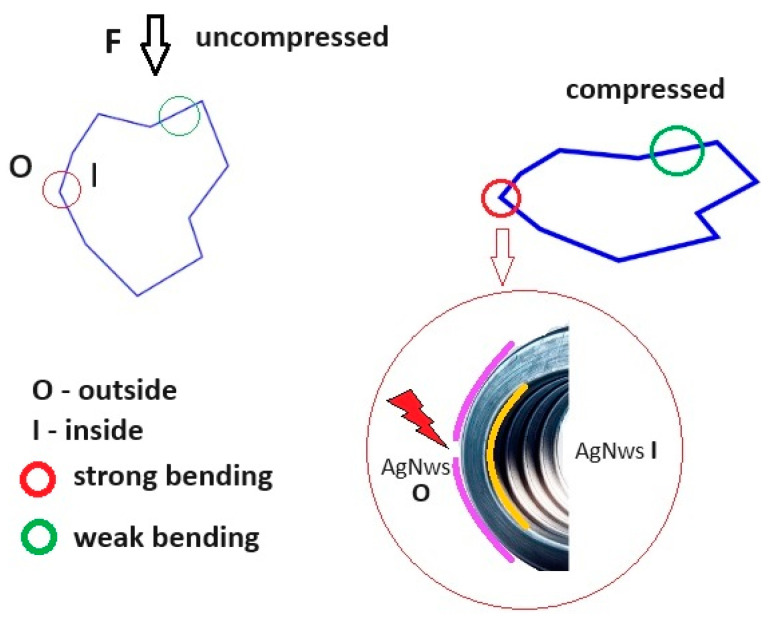
Section through an asymmetric pore undergoing compressive deformation.

The synthesized material consisted of a PUF with open pores coated with an electroconductive layer of AgNWs. Therefore, there was a significant difference in elasticity between these two materials, with metal foams typically undergoing plastic deformations. Figure 11 presents a possible section through an asymmetric pore undergoing deformation in the direction indicated by the black arrow. During compression, the area marked with a red circle underwent more significant deformation than the area marked with a green circle. Outside the pore, during compression, AgNWs-O were subjected to a stretching force, while inside the pore, AgNWs-I were subjected to a compression force.

Since the AgNWs were not strongly connected to each other outside the pore, the electrical circuit could be interrupted by reducing the number of contact points between the AgNWs. Considering the resistivity of silver as 1.59 × 10^−8^ Ω⋅m, the electrical resistance of a single AgNW with a diameter of 60 nm and a length of 20 μm was 112.5 Ω, as given by the formula:

R = ρL/A(4)

where R is the electrical resistance in ohms (Ω), ρ is the material’s resistivity in ohm·meters (Ω⋅m), L is the length of the conductor in meters (m), and A is the cross-sectional area of the conductor in square meters (m^2^).

Therefore, even in cases where the density of AgNWs on the PUF surface was high, even for samples with a single layer, the initial electrical resistance of the order of tens of kΩ of the sample was primarily determined by the point of electrical contact between the PVP-coated AgNWs. The constant electrical resistance for low degrees of compression indicated a constant number of contact points between the AgNWs.

**Figure 12:**
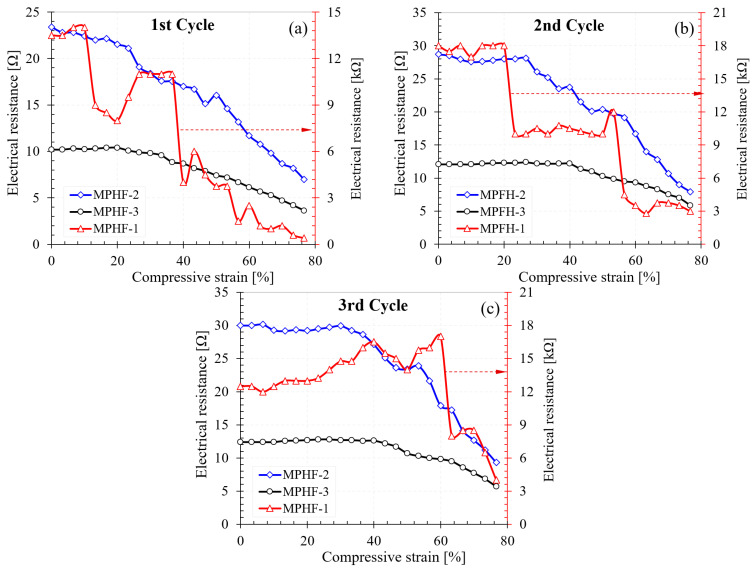
Electrical resistance of the investigated samples for cycle 1 (**a**), cycle 2 (**b**), and cycle 3 (**c**) of compression.

Figure 12 shows a comparison of the electrical resistance of the three samples (MPHF-1, MPHF-1, and MPHF-3) for the three loading cycles. It can be seen that the electrical resistance of the foams coated with metal nanowires decreased with the increase in the amount of AgNWs on the surface of the cells. The electrical resistance of the sample coated with two layers of AgNWs was almost three orders of magnitude lower than that of the sample coated with a single layer. This suggested that, after the first heating of the foams coated with AgNWs at a temperature of 150 °C, the metallic nanowires adhered to the PEO layer deposited on the polymer. The PEO deposited from the solution melted during heating, with its melting temperature being between 66 and 75 °C. By melting, it moistened the PUF surface through the chemical groups (–CH_2_–) involved in van der Waals bonds. However, the hydrophilic character increased through the bonds (–OH) in the PEO structure. As is known, the AgNWs obtained by solvothermal synthesis maintained a layer of PVP strongly adsorbed on the {100} face of the metallic AgNWs through Ag−O coordination [60,61]. This structure allowed for the formation of hydrogen bonds between the (–OH) groups of PEO and the non-participating electron pairs of nitrogen in the PVP structure. The AgNWs fixed after the first thermal treatment on the PUF surface increased the roughness of this surface, facilitating the deposition of a much larger amount of AgNWs after the second immersion in the AgNW suspension. This accelerated deposition mechanism was in agreement with the electrical resistance measurements and SEM images. After the deposition of the third layer of nanowires, the electrical resistance dropped by only 50%, indicating a limitation of the AgNW deposition process. Therefore, the deposition of a new layer would be unjustified from a cost-effectiveness perspective. Another important observation consists of the deviation in the measured values of the electrical resistance from their average during the compression cycles. It was observed that this deviation decreased with the increase in the degree of coating of the surface with AgNWs. Sample MPHF-3 behaved practically like a true ohmic conductor, with the resistance value decreasing almost linearly with the degree of compression between 30 and 80%.

A very interesting phenomenon was observed after two and three cycles of compression, respectively. For the sample coated with a single layer of AgNWs (MPHF-1), the electrical resistance values were unreproducible due to the reduced number of AgNWs involved in the electrical conductivity. For the samples coated with two (MPHF-2) and three (MPHF-3) layers, the decrease in electrical resistance occurred at increasingly higher values of the degree of compression with the increase in the number of compression cycles. Thus, for the MPHF-2 sample, the decrease in electrical resistance was observed throughout the 1st cycle, from a compression of 26.7% in the 2nd cycle and 33.3% in the 3rd cycle. For sample MPHF-3, these values were at compressions of 20.0% at cycle 1, which increased to 40.0% in cycle 2 and 41.6% in cycle 3 of compression. The stepwise decreases and increases in electrical resistance in these samples were mainly conditioned by the number of contact points between wires before reaching the cellular walls. For this reason, the electrical resistance decreased from small degrees of compression, when percolation probably did not exist, because the pore walls were not deformed enough to reach new walls. As the wire density increased, the redistribution of contact points during compression had a lesser influence than the percolation process, which caused the electrical resistance not to decrease until reaching higher degrees of compression, which allowed for the reaching of new cellular walls and the reconfiguration of the electric current path through the foam. The most visible effect was therefore observed after the first cycle of compression and was probably due to the rearrangement of strongly unbonded AgNWs in successive deposited layers. Thus, it can be considered that PUF foams coated with AgNWs have a compression memory effect and could be used as cheap solutions in industrial processes in which high precision is not required, such as exceeding a maximum admissible load or as ohmic seals for evidence openings of enclosures or cases. The term “memory effect” refers to the different electrical response to compression during various compression cycles, mainly due to changes in the number of contact points between AgNWs as a result of compression cycles, as well as the appearance of cracks in the cellular walls of the PUFs.

In certain applications, the material must not withstand multiple deformations. In the work of Banica et al. [48], the authors demonstrated that AgNWs deposited on PUR could be used as gas sensors for the detection of H_2_S, which reacts with the surface of the silver. The embedding of the AgNWs in the polymeric mass reduced their chemical reactivity. In the mentioned work, the authors showed that AgNWs can be decorated with silver dendrites through electrochemical deposition, with these being deposited at the ends and edges of the wires at more electropositive potentials and over the entire surface of the wires, including the {100} crystalline face protected by a PVP adsorbed layer during the synthesis process, at more negative potentials. Embedding the wires in a polymer requires the use of a higher mass percentage of silver to achieve electrical conductivity, allowing the electrochemical deposition of silver dendrites only on the surface of the foam in contact with the silvering solution. The use of these foams in the process of bacteriological air purification can predict hydraulic pressure drops and the porous layer and can correlate them with the electrical resistance of the foam. The hydraulic pressure drop increases with the degree of compression of the foam necessary to reduce the size of the pores of the filter masses. Our proposed solution represents a compromise between the low price at which this foam can be obtained, the particularly easy capacity for metal recycling through the selective dissolution of silver, and resistance to cyclic deformation, which is less necessary in the applications mentioned above.

The price of one gram of high-quality AgNWs in 2024 is approximately 300 USD/g [62]. Considering a specific mass of 100 mg/cm^2^ and a surface area of 1 cm^2^ for a foam with cubic pores having sides of 1.5 mm and foam struts with a circular section of approximately 6 cm^2^ with a diameter of 0.25 mm, the cost of the materials used is about 0.25 USD/cm^3^, and the total manufacturing cost of such a pressure transducer with bactericidal properties is likely less than USD 0.35. The use of lower-quality AgNWs can reduce the price to less than USD 0.2.

## 4. Conclusions

For the first time, flexible open-cell PUF/AgNW (polyurethane foam/silver nanowire) structures have been manufactured and investigated.

It has been demonstrated that polyethylene glycol (PEG) can represent an environmentally friendly solution for improving the hydrophilicity of the PUF surface. Simple immersion of the PUF in 0.1% PEG solution leads to surface activation and good adsorption of AgNWs by creating hydrogen bonds between the hydroxyl groups of PEG and the non-participating electron pairs of PVD adsorbed on the AgNWs. At low coating rates (e.g., 1st immersion), the deposition of AgNWs is inhomogeneous, with non-conductive areas on the PUF surface. Increasing the degree of AgNW coating (e.g., 2nd and 3rd immersion) leads to the formation of a continuous network of electroconductive wires on the foam surface.

The electrical resistance of new-developed metal-polymer hybrid foams (MPHFs) for various degrees of AgNW coating was measured during repeated compression tests. For low degrees of AgNW coating, the decrease in electrical resistance during compression occurs in steps and is not reproducible with repeated compression cycles due to the reduced number of electroconductive zones involved in obtaining electrical conductivity. For high AgNW coating degrees, at foam compressions of 35–80%, the decrease in resistance is quasi-linear and reproducible after the first compression cycle. However, after compression, cracks appear in the foam cell structure that increase the electrical resistance and decrease the mechanical strength.

Thus, the proposed highly electroconductive MPHF structures can provide an alternative for the construction of cheap pressure transducers with limited lifetimes or can be used as filter media for air conditioning units, presenting bactericidal and antifungal properties due to the silver in the structure.

## Data Availability

Data are contained within the article.
